# Increased Abundance of Lactobacillales in the Colon of Beta-Adrenergic Receptor Knock Out Mouse Is Associated With Increased Gut Bacterial Production of Short Chain Fatty Acids and Reduced IL17 Expression in Circulating CD4^+^ Immune Cells

**DOI:** 10.3389/fphys.2018.01593

**Published:** 2018-11-13

**Authors:** Akeem Bartley, Tao Yang, Rebeca Arocha, Wendi L. Malphurs, Riley Larkin, Kacy L. Magee, Thomas W. Vickroy, Jasenka Zubcevic

**Affiliations:** Department of Physiological Sciences, College of Veterinary Medicine, University of Florida, Gainesville, FL, United States

**Keywords:** microbiota, IL17, colon, sympathetic nervous system, adrenergic receptors

## Abstract

Emerging evidence suggests an associative link between gut dysbiosis, the autonomic nervous system (ANS) and the immune system in pathophysiology of neurogenic hypertension (HTN). However, the close interplay between these three systems presents us with difficulties in deciphering the cause-effect relationship in disease. The present study utilized beta 1 and 2 adrenergic receptor knock out (AdrB1^tm1Bkk^AdrB2^tm1Bkk^/J KO) mice to isolate the effects of reduced overall sympathetic drive on gut microbiota and systemic immune system. We observed the following: (i) Diminished beta adrenergic signaling mainly reflects in shifts in the Firmicutes phyla, with a significant increase in abundance of largely beneficial Bacilli Lactobacillales in the KO mice; (ii) This was associated with increased colonic production of beneficial short chain fatty acids (SCFAs) butyrate, acetate and propionate, confirming functional microbiota shifts in the KO mice; (iii) Dampened systemic immune responses in the KO mice reflected in reduction on circulating CD4^+^.IL17^+^ T cells and increase in young neutrophils, both previously associated with shifts in the gut microbiota. Taken together, these observations demonstrate that reduced expression of beta adrenergic receptors may lead to beneficial shifts in the gut microbiota and dampened systemic immune responses. Considering the role of both in hypertension, this suggests that dietary intervention may be a viable option for manipulation of blood pressure via correcting gut dysbiosis.

## Introduction

Hypertension (HTN), or high blood pressure (BP), is one of the most modifiable risk factors of cardiovascular disease (CVD), which is among the leading causes of death in the United States. HTN is regarded as the “silent killer” as there are no obvious symptoms until development of overt clinical signs. Untreated or uncontrolled HTN typically results in end-organ damage such as hemorrhagic stroke, heart disease, peripheral arterial disease, retinopathy, and renal failure (Chobanian et al., 2003). A significant proportion of all HTN cases are either resistant or refractory to currently available antihypertensive treatments, and many of them have no clear etiology, thus characterized by primary or essential HTN (Calhoun et al., 2008). One of the characteristics of essential HTN is its neurogenic component, reflecting in overactivation of the sympathetic nervous system (SNS) that is involved in initiation and maintenance of elevated BP via activation of adrenergic receptors (Esler et al., 1981; Anderson et al., 1989; Rumantir et al., 2000; Schlaich et al., 2004). In addition, evidence links rodent models of neurogenic HTN with exaggerated SNS activity to chronic low-grade inflammation and neuronflammation (Harrison, 2014; Singh et al., 2014; Zubcevic et al., 2014), and most recently gut dysbiosis (Yang et al., 2015, 2017; Adnan et al., 2017; Raizada et al., 2017; Santisteban et al., 2017).

Human gastrointestinal (GI) tract provides one of the best examples of symbiosis in nature. The gut represents a complex ecosystem of host-bacteria interaction comprised of approximately 100 trillion bacteria (Chow et al., 2010). Despite the dynamic range of bacteria present on earth, the gut microbial community is mainly dominated by four phyla: (1) Firmicutes, (2) Bacteroidetes, (3) Actinobacteria and (4) Proteobacteria (Chow et al., 2010). Existing studies have illustrated that gut microbiota has a profound impact on host biology ranging from systemic immunity and morphological integrity of intestinal epithelial cells, to endocrine and absorptive functions (Bry et al., 1996; Hooper et al., 2001; Pamer, 2007; Smith et al., 2007; Round and Mazmanian, 2009; Chow et al., 2010). In order to establish a healthy steady state, host-microbiota interactions require a delicate balance (Sansonetti, 2004; Pamer, 2007; Smith et al., 2007), with regulatory mechanisms in place that serve to regulate bacterial colonization of the gut, while simultaneously enabling immune responses against harmful antigens (Chow et al., 2010).

There is no absolute definition of a “healthy” gut microbiota as yet, however, evidence suggests that the ratio of Firmicutes (F) to Bacteroidetes (B), the F/B ratio as a biomarker for pathological conditions (Mariat et al., 2009; Sanz and Moya-Pérez, 2014; Yang et al., 2015, 2017; Adnan et al., 2017; Santisteban et al., 2017). Recent data suggests that changes in gut microbiota might also impact disorders such as metabolic syndrome, diabetes, and obesity (Tilg and Kaser, 2011; Everard and Cani, 2013), as well as neurogenic diseases and conditions including but not limited to anxiety, sleep apnea, and HTN, all marked by significantly altered sympathetic activity (Cryan and Dinan, 2012; Zubcevic et al., 2014; Mell et al., 2015; Yang et al., 2015, 2017; Adnan et al., 2017; Santisteban et al., 2017). To defend the host against pathologic threats and maintain physiological homeostasis, the SNS and immune system are reciprocally regulated (Bellinger and Lorton, 2014; Jänig, 2014). Moreover, the host-microbiota interaction also reflects in direct communication between the gut bacteria and the host immune system, and dysregulation of the immune system has been associated with intestinal diseases such as colorectal cancer and inflammatory bowel disease (Baumgart and Carding, 2007; Gao et al., 2015). Given the regulatory role of the SNS on immune system and BP (Zubcevic et al., 2014; Yang et al., 2017) and the presence of gut dysbiosis and exaggerated immune system in conditions such as HTN (Santisteban et al., 2015, 2017; Yang et al., 2015; Adnan et al., 2017), we hypothesized that blockade of the effects of sympathetic drive will result in beneficial changes in the immune system responses and shifts in the gut microbiota richness and diversity, leading to functional changes such as production of gut microbiota metabolites. To test our current hypothesis, we utilized beta 1 and beta 2 adrenergic receptor (AdrB1^tm1Bkk^AdrB2^tm1Bkk^/J) knockout (b1.b2 Adr KO) mice, characterized by reduced BP and heart rate responses (Rohrer et al., 1999) resulting from reduction of the effects of the SNS on the whole body system including the gut and the immune system. Our findings support a role for SNS in modulation of gut microbiota and mainly that of the Firmicutes phylum. Moreover, we note a beneficial reduction in circulating CD4^+^.IL17^+^ T cells and increase in circulating young neutrophils in the b1.b2 Adr KO mice, both previously linked with shifts in the gut microbiota (Zhang et al., 2015; Wilck et al., 2017). Thus, the current may be useful in explaining some of the HTN and similar inflammatory phenotypes.

## Methods

### Animal models

All experimental procedures were approved by the University of Florida Institutional Animal Care and Use Committee and complied with the standards stated in the National Institutes of Health Guide for the Care and Use of Laboratory Animals. Mice were housed in a temperature-controlled room (22°C to 23°C) with a 12:12-h light-dark cycle, in specific-pathogen free cages, and had access to standard mice chow and water *ad libitum*. Twenty-week old male heterozygous beta 1 and beta 2 adrenergic receptor (AdrB1^tm1Bkk^AdrB2^tm1Bkk^/J) knockout (KO) mice (#003810, Jax Labs) and their wild type (WT) C57BL/6J controls (#000664, Jax Labs) were used in all experiments. Genoytyping was performed prior to all experiments to confirm the strain differences, as per the Jacksonville Laboratories protocols. Briefly, mouse tails were snipped and lysed in 200 μl of Direct PCR Lysis Reagent (Viagen Biotech cat#101-T) supplemented with 4 μl of 200 mg/ml Proteinase K solution (Fisher cat#BP1700-100). Following 6 h of incubation at 55°C, and additional 45 min incubation at 85°C, the samples were briefly centrifuged and the DNA lysate was used for PCR. The following primers were used in regular PCR reaction, as per The Jackson Laboratory protocol for Adrb1^tm1Bkk^-Probe (see https://www2.jax.org/protocolsdb/f?p=116:1:0::NO:RP::) (i) to confirm the KO strain: F: TTG GGA AGA CAA TAG CAG GC, and R: CGC TGT CCA CAG TGG TTG T; (ii) to confirm the wild type strain: F: ATC TGG TCA TGG GAT TGC TG, and R: ACA CAG CAC ATC TAC CGA AG. The PCR reaction was performed using Amplitaq Gold 360 Master Mix (Invitrogen cat#4398876), and products were run on a 1% agarose gels. Using this protocol, the KO strain showed one band at 113 bps, while the wild type strain showed two bands, one at 113 bps, and one and 225 bps, as per the Jacksonville Laboratories instructions.

### RNA extraction, cDNA preparation, and real time PCR (qPCR) in colon of WT and KO mice

Strains were confirmed by genotyping as per The Jackson Laboratory protocols prior to commencement of experiments. RNA extraction was performed as previously described (Yang et al., 2017). Briefly, extraction of RNA from proximal/distal colon (*n* = 7 per strain) was performed using 1mL TRIzol® Reagent (#15596018, Life Technologies, Carlsbad, CA), as per manufacturer's protocol. Final RNA pellets were resuspended in 30μL of RNAse-DNAse free water to avoid potential genomic contamination. RNA concentration was determined using the NanoDrop-2000 spectrophotometer (Thermo Scientific), then subsequently diluted to 100 ng/μL. After dilution, 1 μg of RNA was used for cDNA synthesis using the High Capacity cDNA Reverse Transcriptase Kit (#4368814, Thermo Scientific). PCR was performed to evaluate the expression of beta 1 and 2 adrenergic receptors (B1AdrR: *forward* GAA GGC GCT CAA GAC ACT GG; *reverse*: CCA GGT CGC GGT GGA A; B2AdrR: *forward* ATG TCG GTT ATC GTC CTG GC; *reverse*: AAG TCC AGA ACT CGC ACC AG) using the CFX96 Touch™ Real-Time PCR Detection System (Bio-Rad, Hercules, California). Two additional transcripts were analyzed in order to evaluate potential presence of overall inflammatory markers in the colon: TNFalpha (TNF-α; *forward*: AGT GGT GCC AGC CGA TGG GTT GT; *reverse*: GCT GAG TTG GTC CCC CTT CTC CAG) and IL-6 (*forward*: AAA GAG TTG TGC AAT GGC AAT TCT; *reverse*: AAG TGC ATC ATC GTT GTT CAT ACA). The conditions of the PCR were as follows: 95°C for 3 min, followed by 40 cycles of 95°C for 10 s, primer annealing at 60°C for 30 s, and 72°C for 30 s. Dissociation curves were generated, starting at 75°C and ending at 95°C with increments of 0.5°C every 5 s, to confirm the specificity of reaction. Results were analyzed using the 2-ΔCt method as previously described (Zubcevic et al., 2017) by normalizing to the housekeeping gene GAPDH (*forward*: GGT GAA GGT CGG TGT GAA CG; *reverse*: CTC GCT CCT GGA AGA TGG TG), as an endogenous control. Additional no reverse transcriptase control was used.

### Fecal DNA extraction from colon of WT and KO mice

All mice were euthanized with 4% isoflurane in a mixture of 95%/5% of O_2_/CO_2._ Fecal samples were collected from the proximal and distal colon region from all WT and KO mice separately at end point (*n* = 5–6 per strain). Bacterial DNA was extracted using ZR Fecal DNA MiniPrep (Zymo Research, Irvine, CA) as per manufacturer's protocols. DNA concentrations were determined using the NanoDrop-2000 spectrophotometer (Thermo Scientific).

### 16 rDNA sequencing and analyses

The analyses of microbial composition were performed, as previously described (Yang et al., 2015, 2017; Santisteban et al., 2017). Briefly, bacterial 16S rDNA V4-V5 regions were obtained using Illumina Miseq compatible primers (Table 1). PCR amplicons were purified (Qiagen, Madison, WI) following separation on an agarose gel, and quantified by the Qubit Fluorometer (Invitrogen, Grand Island, NY). Equal amounts of amplicon for each sample were qualified with Kapa SYBR fast qPCR kit (Kapa Biosystems, Inc., Woburn, MA). Individual samples were run as a library on the Illumina Miseq platform using Miseq v2 reagent kit (Illumina, Inc., San Diego, CA). Primers used for amplification are listed in Table 1. The bioinformatics analyses were performed using QIIME 1.9.2 as described previously (Yang et al., 2015, 2017; Santisteban et al., 2017). Briefly, sequenced reads were demultiplexed according to a combination of forward and reverse indices. Subsequently, obtained reads were filtered based on additional requirements: exact match to sequencing primers and quality score of 30 or more. Filtered reads were applied against Greengene database, assigned taxonomic classifications at a 97% clustering threshold, and summarized in a refOTU table. Alpha, beta diversity, and principal coordinate analyses (PCoA) measures were created using QIIME. Linear discriminant analysis (LDA) along with effect size measurements (LEfSe) in Galaxy was used to distinguish differentially significant features at each taxonomic level. Bacterial genera were identified based on 16S sequencing data (Yang et al., 2015, 2017; Santisteban et al., 2017).

**Table 1:** Primer sequences for microbiota 16S V4-5 amplification.

**Name**	**Forward primer**	**Reverse primer**
C57 WT1	AATGATACGGCGACCACCGAGATCTACACTCTTTCCCTACACGACGCTCTTCCGATCTTAGCTGCCAGCMGCCGCGGTAAT	CAAGCAGAAGACGGCATACGAGATCGGTCTCGGCATTCCTGCTGAACCGCTCTTCCGATCTGTAGCACCGTCAATTYYTTTRAGTTT
C57 WT2	AATGATACGGCGACCACCGAGATCTACACTCTTTCCCTACACGACGCTCTTCCGATCTAGCTACCCAGCMGCCGCGGTAAT	CAAGCAGAAGACGGCATACGAGATCGGTCTCGGCATTCCTGCTGAACCGCTCTTCCGATCTGTAGCACCGTCAATTYYTTTRAGTTT
C57 WT3	AATGATACGGCGACCACCGAGATCTACACTCTTTCCCTACACGACGCTCTTCCGATCTAGCTACCCAGCMGCCGCGGTAAT	CAAGCAGAAGACGGCATACGAGATCGGTCTCGGCATTCCTGCTGAACCGCTCTTCCGATCTCACTGTCCGTCAATTYYTTTRAGTTT
C57 WT4	AATGATACGGCGACCACCGAGATCTACACTCTTTCCCTACACGACGCTCTTCCGATCTAGCTACCCAGCMGCCGCGGTAAT	CAAGCAGAAGACGGCATACGAGATCGGTCTCGGCATTCCTGCTGAACCGCTCTTCCGATCTATTGGCCCGTCAATTYYTTTRAGTTT
C57 WT5	AATGATACGGCGACCACCGAGATCTACACTCTTTCCCTACACGACGCTCTTCCGATCTAGCTACCCAGCMGCCGCGGTAAT	CAAGCAGAAGACGGCATACGAGATCGGTCTCGGCATTCCTGCTGAACCGCTCTTCCGATCTGATCTGCCGTCAATTYYTTTRAGTTT
b1.b2 Adr KO1	AATGATACGGCGACCACCGAGATCTACACTCTTTCCCTACACGACGCTCTTCCGATCTTAGCTGCCAGCMGCCGCGGTAAT	CAAGCAGAAGACGGCATACGAGATCGGTCTCGGCATTCCTGCTGAACCGCTCTTCCGATCTTACCATCCGTCAATTYYTTTRAGTTT
b1.b2 Adr KO2	AATGATACGGCGACCACCGAGATCTACACTCTTTCCCTACACGACGCTCTTCCGATCTAGCTACCCAGCMGCCGCGGTAAT	CAAGCAGAAGACGGCATACGAGATCGGTCTCGGCATTCCTGCTGAACCGCTCTTCCGATCTCGTGATCCGTCAATTYYTTTRAGTTT
b1.b2 Adr KO3	AATGATACGGCGACCACCGAGATCTACACTCTTTCCCTACACGACGCTCTTCCGATCTAGCTACCCAGCMGCCGCGGTAAT	CAAGCAGAAGACGGCATACGAGATCGGTCTCGGCATTCCTGCTGAACCGCTCTTCCGATCTACATCGCCGTCAATTYYTTTRAGTTT
b1.b2 Adr KO4	AATGATACGGCGACCACCGAGATCTACACTCTTTCCCTACACGACGCTCTTCCGATCTAGCTACCCAGCMGCCGCGGTAAT	CAAGCAGAAGACGGCATACGAGATCGGTCTCGGCATTCCTGCTGAACCGCTCTTCCGATCTGCCTAACCGTCAATTYYTTTRAGTTT
b1.b2 Adr KO5	AATGATACGGCGACCACCGAGATCTACACTCTTTCCCTACACGACGCTCTTCCGATCTAGCTACCCAGCMGCCGCGGTAAT	CAAGCAGAAGACGGCATACGAGATCGGTCTCGGCATTCCTGCTGAACCGCTCTTCCGATCTTCAAGTCCGTCAATTYYTTTRAGTTT
b1.b2 Adr KO6	AATGATACGGCGACCACCGAGATCTACACTCTTTCCCTACACGACGCTCTTCCGATCTAGCTACCCAGCMGCCGCGGTAAT	CAAGCAGAAGACGGCATACGAGATCGGTCTCGGCATTCCTGCTGAACCGCTCTTCCGATCTCTGATACCGTCAATTYYTTTRAGTTT

### Short chain fatty acid (SCFA) extraction and high performance liquid chromatography (HPLC) for determination of fecal SCFA levels

SCFAs were extracted from colonic fecal pellets from all mice (*n* = 4/strain) as previously described (Yang et al., 2017). Briefly, 500 μL of 12M HCl was added to fecal homogenates to preserve the volatile SCFAs. Samples were then vortexed vigorously to suspend the fecal mass. SCFAs were extracted using 5 mL of methylene chloride with gentle rotation at room temperature for 20 min. The supernatant was discarded after centrifugation at 3,500 rpm for 5 min. 500 μL of 1M NaOH were added to the organic pellets, samples were rotated for an additional 20-min at room temperature. Following centrifugation at 3,500 rpm for 5 min, the top aqueous layer was collected and mixed with 100 μL of 12M HCl before being filtered for injection. Standard curves were generated using serial dilutions prepared for each SCFA separately in a similar manner. For example, a 1M stock of sodium butyrate was prepared by dissolving 0.55g of sodium butyrate (#303410-100g, Sigma-Aldrich) into 5 mL of molecular grade water. The solution was then serially diluted to 1 mM. A 1 μM sodium butyrate standard was prepared by mixing 1.5 μL of the 1 mM sodium butyrate stock with 500 μL of molecular grade water and 1000 μL of methanol. A blank standard was also prepared by mixing 500 μL of molecular grade water with 1000 μL of methanol. Each injection used a 50 μL aliquot. The chromatographic separation was performed at room temperature using Perkin Elmer Series 200 HPLC System (Perkin Elmer Instruments, Norwalk, CT), equipped with an autosampler, quaternary pump, and 200 series UV/VIS detector.

### Flow cytometry in blood of WT and KO mice

Flow cytometry analysis was performed in all mice (*n* = 6/strain) to quantify the levels of circulating inflammatory cells (ICs) as previously described (Ahmari et al., 2016; Yang et al., 2017). Briefly, total mononuclear cells (MNCs) were isolated from blood and prepared in a concentration of 0.5-1 × 10E6 cells/100 ul in PBS+2% FBS+1mM EDTA mixture media. Cells were incubated with antibodies in a 1:250 ratio, for 30 min at 4°C. The following markers were used to identify specific inflammatory cells: (i) CD4^+^IL17^+^ (T cells; #100406, BioLegend®; #559502, BD Biosciences), (ii) CD8a (T cells; #553033, BD Biosciences), (iii) CD3e (T cells; #553067, BD Biosciences), (iv) CD11b (monocyte/macrophages; #101205, BioLegend®), (v) Ly6^+^CD184^+^CD62L^+^ (neutrophils; #551460, BD Biosciences; #551966, BD Biosciences; #553152, BD Biosciences). In the case of IL17, which is an intracellular marker, following incubation with anti-CD4, cells were washed in normal PBS and fixed in 1% PFA. Following this, cells were incubated with 0.5% Tween20 in normal PBS for 1 h at RT, in order to permeabilize the cell membrane and allow penetration of anti-IL17 inside the cell. Following this, cells were washed in normal PBS, and incubated with anti-IL17 at 1:250 ratio for 30 min at 4°C. SYTOX blue cell stain (ThermoFisher Scientific, #S34857) was added to all samples per manufacturer protocol for exclusion of dead cells during flow cell analysis. Following incubation with antibodies, the cells were washed in normal PBS and fixed with 1% PFA, apart from IL17-stained samples, which were processed as described above. All samples were read on an LSR-II (BD Biosystems) in University of Florida Interdisciplinary Center for Biotechnology Research (ICBR) and the data were analyzed with FACS Diva software, version 6.1.2. Fractions of young (Ly6^+^CD184^+^CD62L^hi^) and old (Ly6^+^CD184^+^CD62L^lo^) neutrophils was determined as previously described (Zhang et al., 2015).

### Statistical methods

Statistical analysis was performed using GraphPad Prizm and two-tailed unpaired *T*-test was used for comparison between the two experimental groups. *P* < 0.05 was considered significant. In addition, linear discriminant analysis (LDA) along with effect size measurements (LEfSe) in Galaxy and ANOSIM in R were used to distinguish differentially significant features at each taxonomic level.

## Results

### Confirmation of beta adrenergic gene downregulation in the colon of KO mice

To confirm the relative expression levels of beta 1 adrenergic receptors in colon of WT and KO mice, we performed quantitative RT PCR. We observed a significant downregulation in beta 1 adrenergic receptor relative expression levels (Mean ± SEM: 1 ± 0.237 (WT) and 0.289 ± 0.018 (KO); *p* < 0.05; Table 2) and beta 2 adrenergic receptor relative expression levels (Mean ± SEM: 0.0045 ± 0.0001 (WT) and 0.00085 ± 0.00026 (KO); *p* < 0.05; Table 2) in the colon of KO compared to WT mice.

**Table 2:** Relative expression levels of genes in proximal colon of WT C57 and b1.b2 Adr KO mice as obtained by real time qPCR.

	**C57WT**	**b1.b2 Adr KO**	***P*-value**
B1AdrR	1 ± 0.237	^*^0.289 ± 0.018	^*^P < 0.05
B2AdrR	0.0045 ± 0.0001	^*^0.00085 ± 0.00026	^*^P < 0.05
TNF-α	0.0007798 ± 0.0003504	0.0009475 ± 0.0003198	ns
IL-6	0.00006678 ± 0.00003039	0.0001558 ± 0.00004057	ns

* *P < 0.05 vs C57 WT*.

### Relative expression levels of TNF-α and IL-6 in the colon of WT vs. KO mice

We performed quantitative RT PCR to assess the relative gene expression levels of select inflammatory genes in the colon of WT and KO mice (Table 2). Both cytokines have previously linked to proinflammatory responses in the gut and subsequent shifts in the microbiota diversity (Rosser et al., 2014; Jones-Hall et al., 2015; Burton et al., 2017). We observed no significant differences in relative expression of either of the genes investigated: (i) TNF-α: 0.0007798 ± 0.0003504, *n* = 5 (WT) vs. 0.0009475 ± 0.0003198 (KO) (Table 1); (ii) IL-6: 0.00006678 ± 0.00003039, *n* = 7 (WT) and 0.0001558 ± 0.00004057 (KO) (Table 2).

### Alterations in the gut microbiota reflect in increased abundance of bacilli class of firmicutes in the KO mice

**Figure 1:**
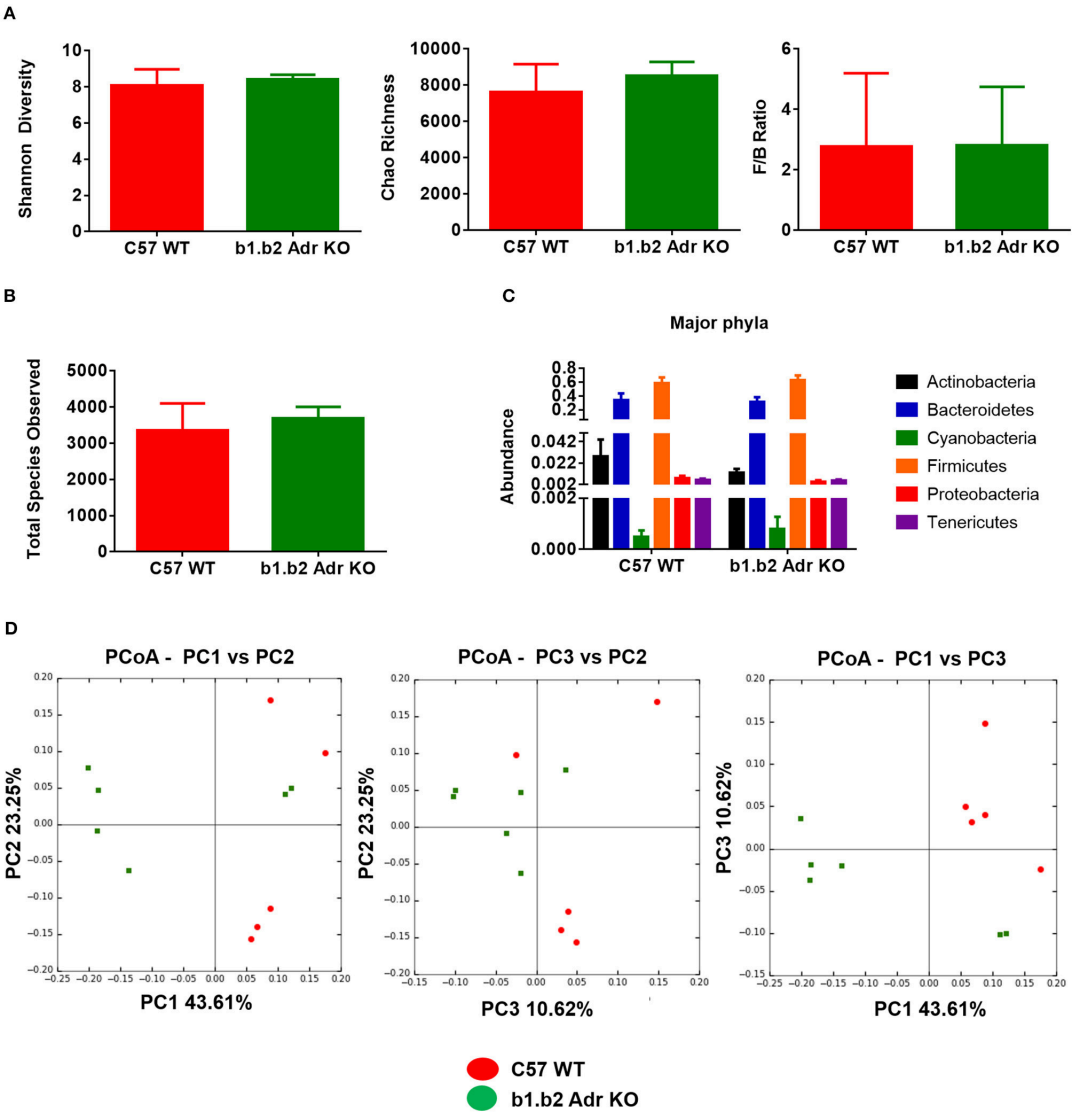
Analysis of gut microbiota in b1.b2 Adr KO and C57 WT mice. **(A)** No significant difference in gut microbiota richness, diversity, or F/B ratios between the KO and WT mice. **(B)** No significant differences in the total bacterial species observed in the KO and WT mice. **(C)** No significant difference in abundance of major bacterial phyla. **(D)** Unweighted Principal coordinate analysis (PCoA) shows a significant separation of red and green clusters representing the WT and KO mice groups, respectively (ANOSIM in R, *P* < 0.05 vs. C57 WT). *N* = 5–6 per strain.

**Figure 2:**
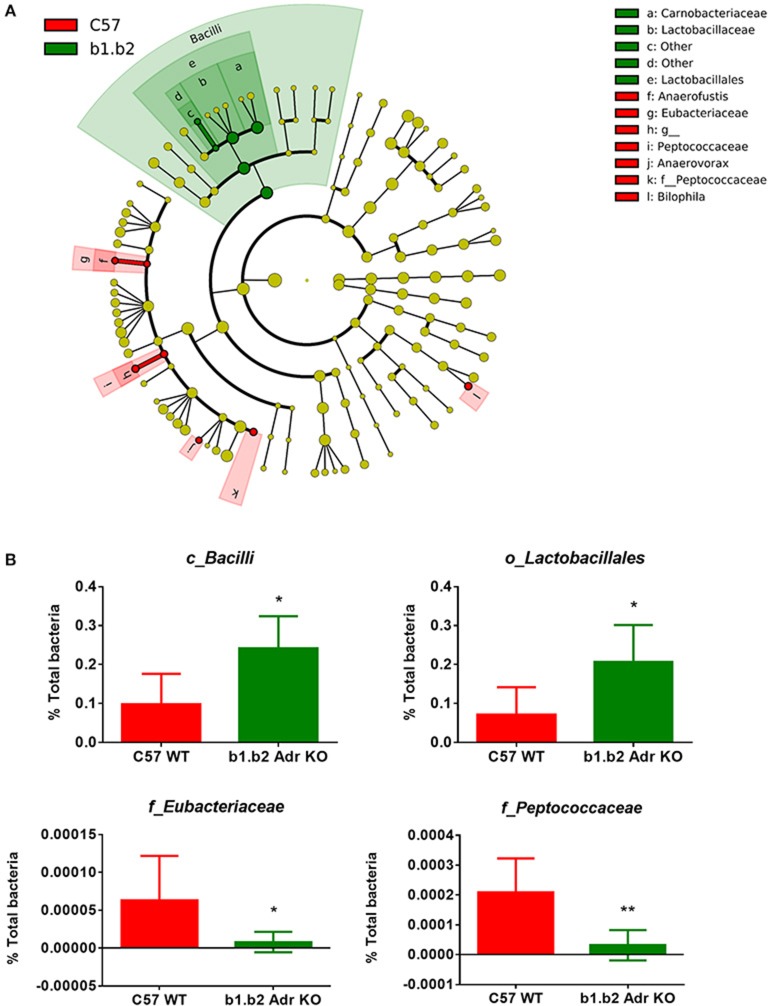
Expansion of Bacilli class of the Firmicutes phylum in the b1.b2 Adr KO mice**. (A)** Taxonomic cladogram was generated by performing linear discriminant analysis (LDA) of effect size (LEfSe) in Galaxy. Each circle dot represents a bacterial taxon with its diameter proportional to the taxon's relative abundance. Differences are represented in colors where red indicates significantly higher abundance in the WT mice, and green indicates significantly higher abundance in the KO mice (*N* = 5–6 per strain). Yellow indicates no significant differences between the two strains. **(B)** Unpaired two-tailed *T* test analyses of specific class (c_), order (o_) and family (f_) from the Firmicutes phyla, with significant differences between the KO and WT mice. *N* = 5–6 per strain, *P < 0.05 and **P < 0.01 vs. C57 WT.

The rodent gut microbiota is generally dominated by Firmicutes and Bacteroidetes phyla, with Proteobacteria, Actinobacteria, and Tenericutes constituting the remaining phyla (Yang et al., 2015, 2017; Santisteban et al., 2017). In our sample, we also observed a small population of Cyanobacteria (Figure 1C). Shifts in the bacterial proportions and phylotypes are widely used to evaluate gut homeostasis. Our 16S rRNA amplicon sequencing data suggested that Chao richness and Shannon diversity indices did not differ between C57 and b1/2-ARs KO mice (Figure 1A), indicating that deficiency in b1/2-AdrRs signaling did not induce significant changes in the microbiota species richness or biodiversity. In addition, we did not observe any difference in the Firmicutes to Bacteroidetes (F/B) ratios (Figure 1A, far right panel) or total species observed (Figure 1B) between WT and KO mice, indicating absence of dysbiosis in the KO strain. Moreover, there were no significant differences at phylum level between the two strains (Figure 1C). However, analyses of gut microbial community structure by unweighted UniFrac principal coordinate analysis (PCoA) revealed two separate clusters, indicating dissimilarities in microbial community structure between the KO and WT mice (Figure 1D, *P* < 0.05). Analysis of taxonomy indicated a significant increase in the abundance of Bacilli class of Firmicutes, and more specifically in the order of Lactobacillales, with significantly increased abundance of families of Lactobacillaeae and Carnobacteriaceae in the KO compared to WT mice (Figure 2, in green). Moreover, a depletion of Anaerofustis genera of Eubacteriaceae family, the Peptococcaceae family, and Anaerovorax genera, all of Firmicutes phyla, were also observed in the KO vs. WT mice (Figure 2, in red). Thus, overall deficiency in beta adrenergic signaling led to distinct alterations in specific gut Firmicutes with no significant dysbiosis.

### Increased levels of bacterial short chain fatty acid (SCFA) metabolites in feces of KO mice

**Figure 3:**
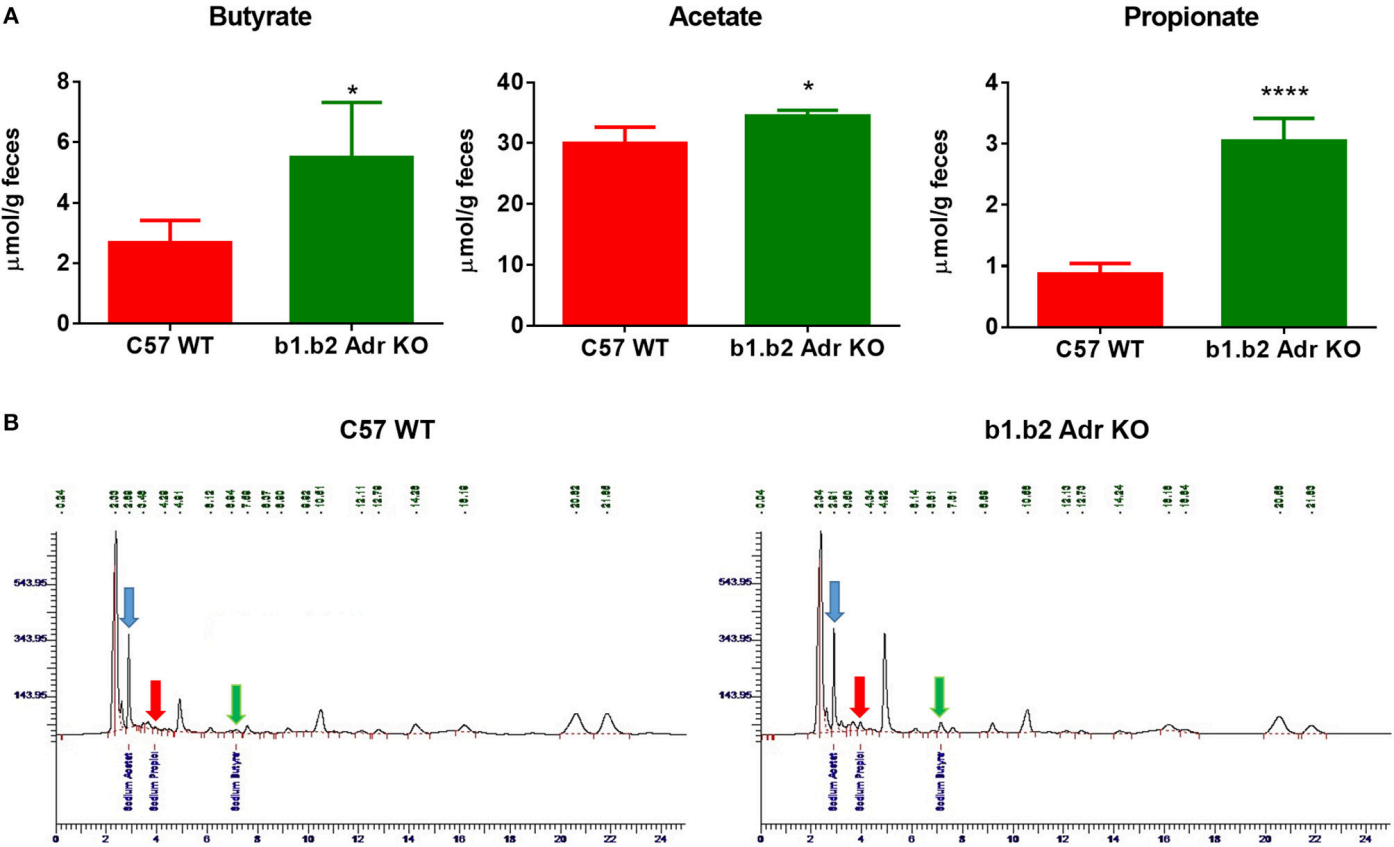
Relative amounts of SCFAs in fecal samples from C57 WT and b1.b2 Adr KO mice. In **(A)**: significantly higher levels of sodium butyrate (left panel), acetate (middle panel) and propionate (right panel) were observed in the KO vs. WT mice. In **(B)**: representative images of butyrate peaks (marked by green arrows), acetate peaks (marked by blue arrows), and propionate peaks (marked by red arrows) in the C57 WT (left panel) and b1.b2 Adr KO mice (right panel), as measured by HPLC. *N* = 4 per strain; *P < 0.05 and ****P < 0.0001 vs. C57 WT.

To functionally confirm observed differences in the gut microbiota between the two strains, we performed HPLC analysis in fecal samples to determine levels of different SCFA bacterial metabolites. We observed a significant increase in the levels of fecal sodium butyrate in KO mice compared to WT (mean ± SEM: 2.7 ± 0.4 μmol/g (WT) vs. 5.5 ± 1 μmol/g (KO), *p* < 0.05; Figure 3A). Furthermore, our analysis revealed a significant increase in fecal acetate levels in KO compared to WT mice (mean ± SEM: 29.9 ± 1.4 μmol/g (WT) and 34.5 ± 1.4 μmol/g (KO), *p* < 0.05; Figure 3A). Lastly, we observed a significant increase in fecal propionate levels in KO compared to WT mice (mean ± SEM: 0.8 ± 0.08 μmol/g (WT) vs. 2.2 ± 0.2 μmol/g (KO), *p* < 0.0001; Figure 3A). Figure 3B shows examples of raw data from HPLC analysis. This suggests a potential beneficial functional shift in gut microbiota in the KO mice.

### KO mice exhibit shifts in levels of circulating CD4^+^.IL17^+^ T cells and “young” neutrophils

**Figure 4:**
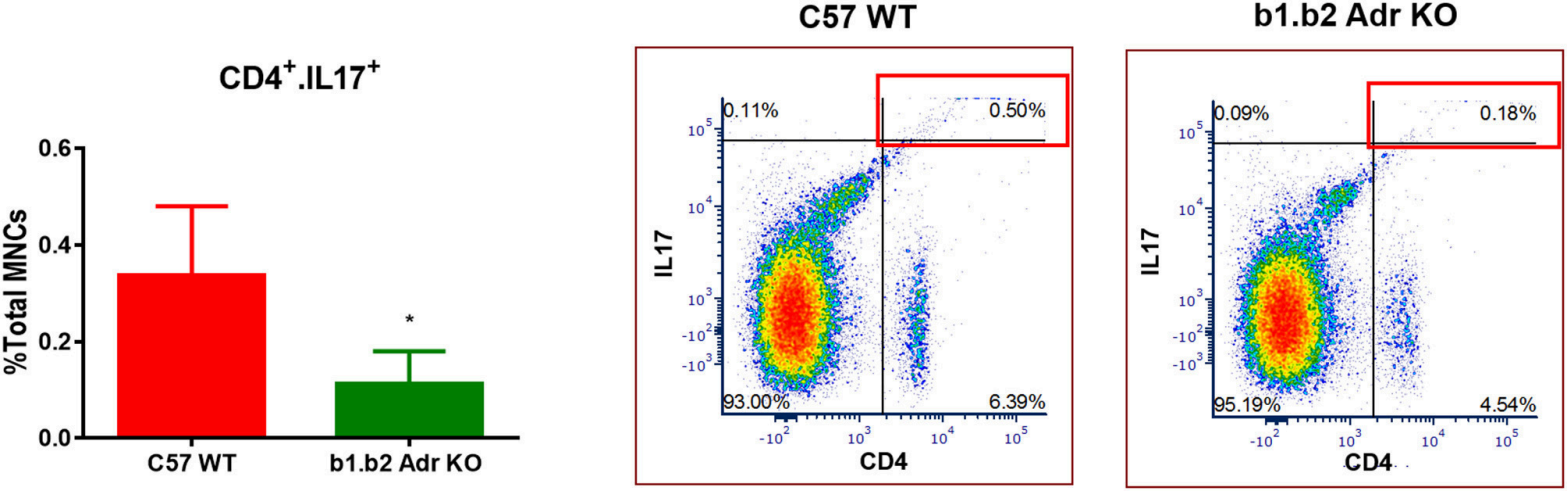
Quantification of circulating inflammatory cells in C57 WT and b1.b2 Adr KO mice. Significantly decreased levels of CD4^+^.IL17^+^ T cells in b1.b2 Adr KO, with representative gating shown in the right panel. *N* = 6 per strain; *p < 0.05 vs. C57 WT.

Flow cytometry analysis was performed to assess the levels of circulating ICs (summarized in Table 2 and Figure 4). Our analysis did not reveal any significant differences in circulating levels of several T cell subtypes: (i) CD3^+^: Mean ± SEM: 0.13637% ± 0.0318% (WT) and 0.1067% ± 0.02028% (KO); (ii) CD8^+^: Mean ± SEM: 4.593% ± 0.6136% (WT) and 5.863% ± 0.4781% (KO); and (iii) CD4^+^: Mean ± SEM: 5.817% ± 0.9342% (WT) and 4.92% ± 0.1943% (KO) (Table 3). Furthermore, we observed no significant differences in levels of circulating CD11b^+^ monocytes/macrophages between the two strains (Mean ± SEM: 10.86% ± 1.468% (WT) and 9.48% ± 0.5786% (KO) (Table 3). However, we observed a significant reduction in circulating levels of CD4^+^.IL17^+^ T cells in the KO mice (Mean ± SEM: 0.34% ± 0.1% (WT) and 0.11% ± 0.06% (KO); *p* < 0.05; Figure 4). Moreover, there was a significant increase in circulating levels of “young” neutrophils (Ly6^+^.CD184^+^.CD62L^hi^) in the KO mice (Mean ± SEM: 29.28% ± 2.349% (WT) and 37.33% ± 0.4447% (KO); *p* < 0.05, Table 3).

**Table 3:** Quantification of circulating inflammatory cells in the KO and WT mice.

	**C57WT**	**b1.b2 Adr KO**	***P*-value**
CD3^+^	0.13637 ± 0.0318	0.1067 ± 0.02028	ns
CD8^+^	4.593 ± 0.6136	5.863 ± 0.4781	ns
CD4^+^	5.817 ± 0.9342	4.92 ± 0.1943	ns
CD11b^+^	10.86 ± 1.468	9.48 ± 0.5786	ns
Ly6^+^.CD184^+^.CD62L^hi^	29.28 ± 2.349	^*^37.33 ± 0.4447	^*^P < 0.05

* *P < 0.05 vs C57 WT*.

## Discussion

Our study highlights important host-microbiota interactions in a mouse model characterized by reduced overall effects of the sympathetic drive. Our major findings are as follows: (i) we observed no major differences in the diversity, richness or F/B ratio in b1.b2 Adr KO mice compared to C57 WT controls, suggesting no presence of gut dysbiosis in these mice. Moreover, there were no differences in the abundance of the major phyla between the two strains. (ii) However, unifrac unweighted PCoA revealed a significant separation between microbiota of C57 WT and b1.b2 Adr KO mice. This was reflected in significantly increased abundance of the Bacilli class of Firmicutes in the b1.b2 Adr KO mice, and specifically the family of Lactobacillaceae. Conversely, we observed an increased abundance of family of Peptococcaceae and Eubacteriaceae of the Firmicutes phyla, among several others, in the b1.b2 Adr KO mice. (iii) The increase in levels of butyrate, acetate and propionate in the feces of b1.b2 Adr KO mice, linked to anti-inflammatory and anti-hypertensive effects (Natarajan and Pluznick, 2014; Natarajan et al., 2016; Ohira et al., 2017; Pluznick, 2017), suggested beneficial functional shifts in the gut microbiota in these mice. (iv) While we observed no significant differences in inflammatory cytokines between the two strains, suggesting no major difference in local gut inflammatory profile, our data does show a reduction in circulating CD4^+^.IL17^+^ T cells in the b1.b2 Adr KO mice, previously linked to rodent and human HTN (Wang et al., 2016; Wilck et al., 2017; Zubcevic et al., 2017; Kim et al., 2018), while an observed increase in the circulating levels of young neutrophils in these mice has also previously been directly linked to beneficial changes in the gut microbiota (Zhang et al., 2015).

Previous studies have characterized HTN with alterations in the gut microbiota and sympathetic dysregulation (Zubcevic et al., 2014; Yang et al., 2015; Adnan et al., 2017; Santisteban et al., 2017). In the present study, we investigated the close association between the SNS, immune system and gut microbiota in a b1.b2 Adr KO mouse model characterized by reduced BP and heart rate responses upon stimulation (Rohrer et al., 1999). This allowed isolation of the effects of one variable, the SNS, in the complex host-microbiota communication in regulation of BP. Our previous data showed that reduced SNS effects specifically on the bone marrow produced a marked immunosuppression and resulted in significant shifts in the microbiota and reduction in BP (Ahmari et al., 2016; Yang et al., 2017). However, these effects were mainly reflected in decreased abundance of specific members of Bacteriodetes (Yang et al., 2017). While an increase in abundance of some members of the Firmicutes was also observed following blockade of bone marrow SNS only (Yang et al., 2017), these changes do not overlap with the microbiota alterations in the full b1.b2 Adr KO mice examined in the present study. Interestingly, a significant decrease in the order of Lactobacillales of the Bacilli family was observed in the spontaneously hypertensive rat (SHR), a rodent model of HTN characterized by both exaggerated SNS and immune system (Zubcevic et al., 2014; Yang et al., 2015; Santisteban et al., 2017). Since this is in exact opposition to our present finding, we propose that the SNS may contribute to regulation of abundance of Lactobacillales order of the Bacilli class. Others have suggested that norepinephrine (NE), one of the neurotransmitters released following stimulation of SNS, can directly modulate bacterial growth (Freestone et al., 2000; Doherty et al., 2009). However, our model is characterized by lack of beta-adrenergic receptors, thus resulting in reduced effects of SNS rather than the inherent differences in SNS innervation or NE release. Thus, the changes in the gut microbiota observed in the b1.b2 Adr KO mice in the present study may be due to modified gut homeostasis resulted from loss of beta-adrenergic receptors. This may reflect in reduced blood supply to the gut, or suppression of the renin-angiotensin-aldosterone system, which could also be a cause of reduced adrenergic receptor activation, and could results in changes in gut microbiota composition. Further studies are required to investigate the role of SNS in gut homeostasis, but previous data from SHR show association between overactive SNS and breakdown in gut epithelium with reduced levels of Lactobacillaceae (Yang et al., 2015; Santisteban et al., 2017). Thus, supplementation with specific Lactobacillales, and especially family of Lactobacillaceae in the SHR may counteract the effects of increased SNS to the gut in this strain. Indeed, previously published research has noted positive cardiovascular effects following supplementation with members of the Lactobacillaceae family or their metabolic products (Sonestedt et al., 2011; Wilck et al., 2017; Hidalgo et al., 2018).

Lactobacillaceae fermentation has been shown to produce powerful bioactive peptides with the capability to inhibit ACE (Fuglsang et al., 2003; Barla et al., 2016), a key enzyme of the renin-angiotensin system (RAS) positively correlated with HTN (Wang et al., 2011; He et al., 2013). Indeed, there has been several clinical studies which have noted reduced BP in hypertensive patients supplemented with specific Lactobacillaceae (Nakajima et al., 1995; Seppo et al., 2003; Jauhiainen et al., 2005; Tanida et al., 2005; Wilck et al., 2017). The increase in fecal butyrate, acetate and propionate confirms a general beneficial shift in the gut microbiota in the b1.b2 Adr KO mice. These short chain fatty acids are metabolite products of gut bacteria with suggested anti-inflammatory and anti-hypertensive properties (Natarajan and Pluznick, 2014; Natarajan et al., 2016; Ohira et al., 2017; Pluznick, 2017). These findings are again in direct opposition to those observed in the SHR, which is characterized by reduced levels of butyrate-, acetate- and propionate-producing gut bacteria (Yang et al., 2015).

Moreover, dietary supplementation with specific members of Lactobacillales has been shown to reduce the effects of increased dietary salt by dampening the IL17-dependent immune responses in rodent models of HTN and human HTN (Wilck et al., 2017). In studies of inflammatory bowel disease, elevated levels of Th17 cells and an upregulation of IL17 have been shown (Chen et al., 2015), and emerging evidence illustrates that certain Lactobacillales strains have the potential to reduce inflammation by modulating IL17 expression (Wells, 2011; Jan et al., 2012; Noto Llana et al., 2013; Wilck et al., 2017). Our present data confirms the positive correlation between elevated abundance of Lactobacillales and reduced CD4^+^.IL17^+^ T cells in circulation of b1.b2 Ard KO mice. Thus, reduced effects of SNS may cause changes in the gut microbiota that may have a direct effect on these immune cells. Evidence arising from experimental models of inflammatory bowel disease suggests that CD4^+^ T cells are key players in the initiation and regulation of the immunopathologic processes, partly through production of proinflammatory cytokines such as tumor necrosis factor-α (TNF-α) (Wells, 2011). Strains of Lactobacillales have also been shown to elicit adaptive and innate immune responses in the host (Noto Llana et al., 2013). However, we did not observe significant differences in expression levels of TNF-α or IL-6 cytokines in the gut of our mice. We also observed a depletion of Eubacteriaceae and Peptococcaceae in the order of Clostridiales and in the phyla of Firmicutes, in our b1.b2 Adr KO mice. These strains are largely considered to be beneficial, with Peptococcaceae a sulfate-reducing bacteria (Petzsch et al., 2015), a property suggested to be important in regulation of BP (Ahmad et al., 2012; Yu et al., 2017). Similarly, we saw a reduction in Bilophila in the b1.b2 Adr KO mice, which are, however considered to be largely pathogenic, which may be related to reduced presence of hydrogen sulfide (da Silva et al., 2008). Further studies are needed to confirm the relevance of these bacterial shifts in cardiovascular and immune homeostasis.

A significant increase in circulating young neutrophils in our b1.b2 Adr KO mice may also be driven by changes in the microbiota in these mice. Neutrophils represent a large population of leucocytes in the mouse, and they have a unique property in that they follow daily cycles of release from the bone marrow into circulation as “young” neutrophils, where their “aging” is positively correlated with pro-inflammatory properties (Casanova-Acebes et al., 2013). Recent work by Zhang et al (Zhang et al., 2015) suggested that neutrophil aging in circulation is regulated by the microbiome, with shifts in abundance of different Bacilli, Clostridiales and Proteobacteria species associated with reduction in circulating aged neutrophils thus dramatically reducing inflammatory organ damage in certain immune disorders (Zhang et al., 2015). Based on this, the observed increase in circulating young neutrophils in our b1.b2 Adr KO mice may be considered anti-inflammatory and microbiota-driven, and may be related to the reduced effects of the SNS. Future data should compare the effects of fecal matter transplant from b1.b2 Adr KO mice on the immune system and SNS, to further characterize role of SNS in the host-microbiota interactions.

In summary, our data illustrates an association between reduced beta-adrenergic receptor signaling, associated with dampened effects of the SNS on the whole body including the gut and the immune system, and a significant shift in Lactobacillaceae family in the gut microbiota and reduced immune responses in mice with reduced BP and heart rate responses (Rohrer et al., 1999). Given the current research interest in the use of probiotics in treating inflammation and gut dysbiosis, our data offers further evidence to why administration of various strains of Bacilli has a positive effect on BP in preclinical and clinical setting.

## Ethics statement

This study was carried out in accordance with the recommendations and approval by the IACUC protocol number 201708217.

## Author contributions

AB and TY designed and performed experiments and analyzed the data. They also contributed to writing of the manuscript. RA, WM, RL, and KM contributed to collection and analysis of data. TV contributed to writing and editing of the manuscript. JZ designed the experiments, oversaw analysis of the data and interpretation of results, and contributed to writing and final editing of the manuscript. Authors would like to thank Niousha Ahmari for technical assistance with the manuscript.

### Conflict of interest statement

The authors declare that the research was conducted in the absence of any commercial or financial relationships that could be construed as a potential conflict of interest.
